# Supplementary material for: Structure-based nuclear import mechanism of histones H3 and H4 mediated by Kap123
Source: eLife. 2017 Oct 16;6:e30244. doi: 10.7554/eLife.30244 (PMC5677370; doi:10.7554/eLife.30244)
Supplement: Supplementary file 1. [file elife-30244-supp1.docx]

**Supplementary Information**

**Structure-based nuclear import mechanism of histones H3 and H4 mediated by Kap123**

Sojin An, Jungmin Yoon, Hanseong Kim, Ji-Joon Song, and Uhn-Soo Cho

**Supplementary Table 1. Data collection and refinement statistics.**

|  | *Kl Kap123* | *Kl Kap123 + Histone H3_1–28_* | *Kl* *Kap123 + Histone H4_1–34_* |
| --- | --- | --- | --- |
| **Data collection** |  |  |  |
| Wavelength (Å) | 0.9786 | 0.9786 | 0.9786 |
| Space group | P1 | P1 | P1 |
| Cell dimensions |  |  |  |
| *a*, *b*, *c* (Å) | 79.05, 88.12, 102.01 | 79.098, 88.114, 102.276 | 78.793, 87.697, 101.623 |
| α, β, γ (°) | 79.19, 80.03, 70.98 | 79.595, 80.771, 70.743 | 79.588, 81.453, 71.699 |
| Resolution (Å) | 48.35 – 2.35 (2.41 – 2.35)* | 50 – 2.70 (2.75 – 2.70) | 50 – 2.82 (2.93 – 2.82) |
| *R*_sym_ or *R*_merge_ (%) | 20.6 (61.5) | 4.3 (58.7) | 4.4 (63.2) |
| *I* / σ*I* | 21.3 (2.5) | 18.8 (1.6) | 20.7 (1.5) |
| Completeness (%) | 99.6 (97.8) | 99.0 (98.3) | 98.9 (98.0) |
| Redundancy | 12.3 | 4.0 | 3.9 |
|  |  |  |  |
| **Refinement** |  |  |  |
| Resolution (Å) | 33.00 – 2.35 (2.41– 2.35) | 50 – 2.70 (2.77 – 2.70) | 33.79 – 2.82 (2.89 – 2.82) |
| No. reflections | 103373 (6754) | 69139 (4652) | 59760 (3762) |
| *R*_work_ / *R*_free_ | 20.99 / 23.49 | 18.50 / 22.97 | 17.02 / 23.39 |
| No. atoms |  |  |  |
| Protein | 15839 | 16047 | 15749 |
| Water | 410 | 82 | 0 |
| *B*-factors |  |  |  |
| Protein | 41.69 | 63.68 | 82.42 |
| Water | 37.06 | 51.31 | n/a |
| R.m.s. deviations |  |  |  |
| Bond lengths (Å) | 0.009 | 0.008 | 0.008 |
| Bond angles (°) | 1.010 | 1.016 | 1.033 |
| Ramachandran plot (%) |  |  |  |
| Favored | 97.82 | 98.14 | 98.55 |
| Outliers | 0 | 0 | 0 |
|  |  |  |  |
| **Protein Data Bank code** | 5VCH | 5VE8 | 5W0V |

* Values in parentheses are for highest-resolution shell.
